# Analytical description of adolescent binge drinking patients

**DOI:** 10.1186/s12887-023-04325-2

**Published:** 2023-10-16

**Authors:** Cordula Haas, Andrea Patrizia Salzmann, Tina Maria Binz, Georg Staubli, Michelle Seiler, Andrea Eva Steuer

**Affiliations:** 1 Zurich Institute of Forensic Medicine, University of Zurich, Zurich, Switzerland; 2 Pediatric Emergency Department and Children’s Research Centre, University Children’s Hospital Zurich, Zurich, Switzerland

**Keywords:** Forensic science, Alcoholism, Binge drinkers, Youth

## Abstract

**Background:**

Binge drinking is a widespread health compromising behavior among adolescents and young adults, leading to significant health problems, injuries and mortality. However, data on alcohol consumption is often unreliable, as it is mainly based on self-reporting surveys. In this five-year study (2014–2019) at the University Children’s Hospital Zurich, we analyzed blood samples from adolescent binge drinking patients to investigate blood alcohol concentrations (BACs), co-ingestion of drugs, assess compliance between self-reported and measured substance use, and test for genetic components of innate alcohol tolerance. Furthermore, hair analysis was performed to retrospectively access drug exposure and to evaluate the potential of hair analysis to assess binge drinking.

**Methods:**

In a prospective, single-center study, patients with alcohol intoxications aged 16 years and younger were included. Blood and hair samples were analyzed by sensitive liquid chromatography – tandem mass spectrometry drug analysis. HTTLPR genotyping was performed with PCR and fragment analysis.

**Results:**

Among 72 cases, 72 blood and 13 hair samples were analyzed. BACs ranged from 0.08–3.20‰ (mean 1.63‰, median 1.60‰), while a mean concentration of 3.64 pg/mg hair (median 3.0 pg/mg) of the alcohol marker ethyl glucuronide (EtG) was detected in eleven hair samples, providing no evidence of chronic excessive drinking. In 47% of the cases, co-ingested drugs were qualitatively detected next to ethanol, but only 9% of the detected drugs had blood concentrations classified as pharmacologically active. Cannabis consumption (22%) and stimulant intake (16%) were the most frequently observed drugs. Compliance between patients’ statements and measured substances matched well. Although we investigated the genetic contribution to innate alcohol tolerance via the 5-HTTLPR polymorphism, the diverse genetic background of the cohort and small sample size did not allow any conclusions to be drawn.

**Conclusion:**

Almost half of our binge drinking patients tested positive for other substances, primarily cannabis. We anticipate that our study enhances understanding of consumption behavior of young people and encourage continued efforts to address the harmful effects of binge drinking and co-occurring substance use.

**Supplementary Information:**

The online version contains supplementary material available at 10.1186/s12887-023-04325-2.

## Background

Binge drinking, also reported as ‘heavy episodic drinking’ (HED) or ‘risky single occasion drinking’ (RSOD), has become a widespread health compromising behavior among adolescents and young adults worldwide [1]. Binge drinking characterizes individuals engaging in excessive (i.e. leading to drunkenness) but episodic alcohol consumption [2]. A binge drinking episode is defined as the consumption of five or more standard drinks for men and four or more standard drinks for women within a 2-hour period or on a single occasion (according to the National Institute on Alcohol Abuse and Alcoholism NIAAA, World Health Organization WHO, Centre for Disease Control CDC) [1]. The definition of a standard drink varies among countries, with a range of 8–20 g of pure alcohol. The repetition of such drunkenness episodes results in cycling between periods of intense alcohol intoxication and abstinence, representing a specific alcohol consumption pattern. Binge drinking is a leading cause of mortality and injury among young adults. It can lead to major health problems, primarily related to alcohol dependence later in life [3], and result in long-term cognitive impairment [4, 5].

Data on alcohol consumption among adolescents and young adults are mainly based on self-reported surveys. Maurage et al. provided an overview of the main tools to assess binge drinking and developed an own evaluation protocol based on six core binge drinking characteristics [2]. However, these questionnaires tend to be unreliable. There is a recall bias with a potential underestimation of alcohol use when recall periods go beyond a couple of days [6]. In addition, underestimation of alcohol consumption increases with heavy consumption [7]. The COMPASS study (Cohort study on Obesity, Marijuana use, Physical activity, Alcohol use, Smoking and Sedentary behavior) was conducted among Canadian high school students and found that current alcohol use was 52–58%, while the binge drinking rate was 34–41% [6]. The international study “Health Behavior in School-aged Children (HBSC)”, conducted in over 40 countries worldwide, reported that in 2015, the weekly alcohol consumption and the binge drinking behavior of 15-year-old Swiss pupils reached the second lowest level since 1986 for boys, while the numbers for females had decreased even further [7]. The percentage of boys who consumed more than five drinks on two or more occasions 30 days prior to the survey declined from 18.6% to 2010 to 14.1% in 2014, while the corresponding percentage for females changed from 15.9 to 12.4%. According to the addiction monitoring in Switzerland in 2023, 33.7% of the 14-15-year-olds consumed alcohol at least once in the last 30 days, while 17.6% engaged in binge drinking at least once a month [8]. However, daily drinking is almost nonexistent in 15-year-olds [9].

Hospitalization due to alcohol intoxication has been reported in children as young as 12–13 years of age [10]. It is likely that these hospitalization numbers represent only a fraction of the true number of cases, since only data from people treated in a hospital were recorded. Therefore, the actual number of cases may be significantly higher. In Swiss hospitals, between 2003 and 2016, 1’231 young people aged 10–23 years were treated for alcohol intoxication or dependency [10]. The number of cases increased by 3% during this period, peaking in 2008, followed by a gradual decrease until 2016. No gender difference in hospitalizations caused by alcohol intoxication was observed up to the age of 15 years, but from the age of 16 years onward, more men than women were treated in hospitals.

In general, adolescents do not drink alcohol habitually and do not acquire alcohol tolerance, making them an ideal study population for investigating innate alcohol tolerance. Acquired tolerance is defined as a reduction in the effects produced by a given dose of a certain substance [11]. The longer the alcohol consumption, the greater the amount required to produce the desired effect. The body develops tolerance in order to be able to function even in an intoxicated condition. In contrast, innate alcohol tolerance affects sensitivity and vulnerability to alcohol. A low level of response (low LR), also referred to as high ethanol tolerance (HET), is a risk factor for the development of an Alcohol Use Disorder (AUD) [12]. Low LR is defined as the need for higher doses of alcohol to produce an effect [13]. Therefore, a person with a low LR or HET is prone to consume more alcohol at a time and heavier drinking is encouraged. Several genes and genetic variants have been identified to contribute to low LR/HET [14–18] and in particular, a variable number of tandem repeats (VNTR) polymorphism (5-HTTLPR) in the serotonin transporter gene *SLC6A4* gene [14, 19]. The repeat is characterized by a 44 bp deletion/insertion leading to a long (L) and a short allele (S) [20]. The S allele is most frequently found in Asians (79%), whereas the L allele is most common in Europeans. Besides these two common variants, there is a rare XL and XS repeat, which is most frequently found in Asians and Africans [21]. Different studies presented different results, either the short (S) or the long (L) form of 5-HTTLPR showed an association with alcohol tolerance and dependence [19, 22–26]. Also, a gene-dose effect was noted, that patients with homozygous genotypes had a greater risk of alcohol dependence compared to those with heterozygous variants [27]. Despite the inconsistent findings in the literature, there may be a trend suggesting that the S allele may be more often associated with alcohol dependence [28]. The association is complex and may vary with the alcohol dependence subtype, the type of drinking behaviour, co-morbid diagnoses, age of onset and ethnicity [28, 29].

The main aim of the present study was to investigate BACs from binge drinking patients and to analyze co-ingestion of drugs or medications other than alcohol. Furthermore, hair analysis was performed in order to retrospectively detect past drug exposure and also to evaluate the potential of hair analysis to assess binge drinking. We also assessed the compliance between the patients’ self-reported substance use and the actual analytical findings in blood and hair samples. Additionally, the 5-HTTLPR polymorphism was examined with the aim to test the influence on innate alcohol tolerance in our cohort of binge drinkers.

## Materials and methods

### Cohort and study design

This prospective, single-center study was performed at the University Children’s Hospital Zurich in collaboration with the University of Zurich. After ethical approval for this study was provided by the local Ethics committee in Zurich (Kantonale Ethikkommission, Ref. KEK-ZH-Nr. 2013 − 0547), enrollments were made from 2014 to 2019 (Table S1).

All patients aged 16 years-old and younger with alcohol intoxication presenting at the emergency department were eligible for this study. Written informed consent was obtained from all parents and children 14 years-old and older, verbal informed consent was obtained from younger patients.

Medical history and co-ingestion of drugs were inquired by medical staff during routine patient treatment. Upon arrival at the emergency department, adolescents were monitored including assessment of the Glasgow Coma Scale (GCS) (motor and verbal response, eye opening) to assess the level of consciousness [30]. Venous blood samples were immediately collected for routine BAC measurements at the hospital diagnostics department (in Heparin coated collection tubes) and for study-specific analyses (in EDTA and Potassium fluoride (KF) coated collection tubes). Hair samples were collected to investigate the long-term consumption of different substances using standard toxicological analysis. However, hair sampling was stopped after a while because patients tended to not participate in the study at all, when they had to provide hair samples, and the hair analysis was initially not a primary focus. Medical history and co-ingestion of drugs were inquired by medical staff during routine patient treatment.

### Sample preparation of whole blood samples for screening and quantitative analysis

KF-stabilized whole blood samples were extracted using protein precipitation (PP) with slight modifications. Details on the method can be found in the Supplementary Material. Subsequently, three separate analyses were conducted: untargeted screening analysis, confirmative, quantitative analysis of a multitude of drugs, and identification and quantification of cannabis, similar to a previous study [31].

### Whole blood (qualitative) screening by LC-MS/MS

Extracted blood samples were analyzed using an untargeted data acquisition approach with a Toxtyper® LC-MS/MS system. Details on the method can be found in the Supplementary Material.

### Quantification of drugs in blood

Quantification of positive results from the blood screening analysis was performed by LC-MS/MS using a targeted in-house multianalyte method. The method covered 82 drugs or drug metabolites from various drug classes such as stimulants, opioids, benzodiazepines, antidepressants, antipsychotics, antitussives, and antihistamines. Limits of quantifications (LOQs) were as follows: 2.4 ng/mL, 6 ng/mL, 2.4 ng/mL, 2.4 ng/mL, 1.6 ng/mL, 8.0 ng/mL, 8.0 ng/mL, 20 ng/mL, 4.0 ng/mL, 2.0 ng/mL, and 16 ng/mL for amphetamine, benzoylecgonine, cocaine, dextromethorphan, diphenhydramine, doxylamine, ketamine, lorazepam, MDMA, methylphenidate, and quetiapine, respectively. Details on the method can be found in the Supplementary Material. The method was fully validated according to national and international guidelines [32–35]. Quantification was performed on the peak area ratios of drug to IS against an eight-point calibration covering an extended therapeutic range for prescription drugs and ranges typically observed in forensic toxicology for drugs of abuse. Each analysis batch was controlled by additional randomized measurements of three quality control samples.

### Quantitative analysis for cannabinoids in blood

Given the undetectability of cannabinoids in the screening analysis, all blood samples were subjected to targeted cannabis analysis on tetrahydrocannabinol (THC), cannabidiol (CBD) and the THC metabolites hydroxy-THC (THC-OH) and THC carboxylic acid (THC-COOH). LOQs were as follows: 0.5 ng/mL, 0.2 ng/mL, 0.5 ng/mL, and 5 ng/mL for THC, CBD, THC-OH, and THC-CCOH, respectively. Sample extracts were analyzed on a Thermo Fischer Ultimate 3000 UHPLC system (Thermo Fischer Scientific) coupled to a Sciex 5500 QTrap linear ion trap quadrupole mass spectrometer (Sciex). Details on the method can be found in the Supplementary Material. MS mode, QC, and data evaluation were performed as described above for the drug quantification.

### Analysis of drugs, medication and ethylglucuronide in hair samples

Thirteen hair samples were collected from the vertex posterior region for analysis. From these samples the proximal 3–5 cm segment was used which represents a time frame to monitor substance intake of 3–5 months. Hair samples were cosmetically untreated. Hair samples were first analyzed for drugs and medication to assess past drug exposure using a multianalyte approach by Scholz et al. [36]. In a second step hair samples that had enough material (> 5 mg) were analyzed for ethylglucuronide. EtG analysis (Limit of detection (LOD) 1 pg/mg and LOQ 2 pg/mg) was performed following the protocol described by Binz et al. [37]. Details on the methods can be found in the Supplementary Material.

### VNTR analysis

DNA was extracted from EDTA blood using the Gentra Puregene Blood core kit A (Qiagen, Hombrechtikon, CH). Primers for the SLC6A4 promotor (5-HTTLPR) [38] were obtained from Microsynth (Balgach, Switzerland). Forward primers contained a FAM modified 5’-end, whereas the reverse primers were unmodified. Amplification of 5-HTTLPR was performed using the GC-RICH PCR-system, dNTPack (Sigma-Aldrich Chemie GmbH, Buchs, Switzerland) as previously described [38]. Fragment length was determined using a 3130xl Genetic Analyzer (Thermo Fisher Scientific) and GeneMapper® ID-X1.4 software (Thermo Fisher Scientific).

To compare the 5-HTTLPR results, binge drinkers were divided into three groups based on their GCS / BAC scores: (1) normal alcohol response: high BAC (drunk) / low GCS (impaired condition) or low BAC (slightly drunk) / high GCS (normal condition); (2) low LR group: high BAC (drunk) / high GCS (normal condition); (3) high LR group: low BAC (slightly drunk) / low GCS (impaired condition). Group 1 showed a normal alcohol response, groups 2 and 3 were regarded as unexpected alcohol responses. The thresholds for grouping were as follows: high BAC ≥ 2‰, low GCS ≤ score 10. Samples where co-ingestion of other drugs were found and explained the low GCS were removed from further analysis. In addition, individuals from non-European or mixed backgrounds were excluded due to potential differences in allele frequencies between different populations. The genetic data of the low and high LR groups were compared.

## Results and Discussion

### Sample cohort and medical history

A total of 72 patients (37 males, 35 females) with a mean age of 14.9 years (SD ± 0.85 years) were included in this study. Of the 72 patients, 51 were of European descent (26 male, 25 female) and 21 were of Non-European or mixed European descent (11 males, 10 females).

Of the 72 patients admitted to the University Children’s Hospital Zurich due to alcohol intoxication, 14 reported having a medical history of various diagnoses and were taking medication regularly. Seven patients had psychiatric and psychological illnesses such as depression, while three participants were treated for attention deficit hyperactivity disorder (ADHD). In addition, six patients reported having asthma, allergies (celiac disease and mite allergy), diabetes, or anemia. An overview of the study patients is provided in Table S1.

### Blood alcohol and EtG

**Fig. 1:**
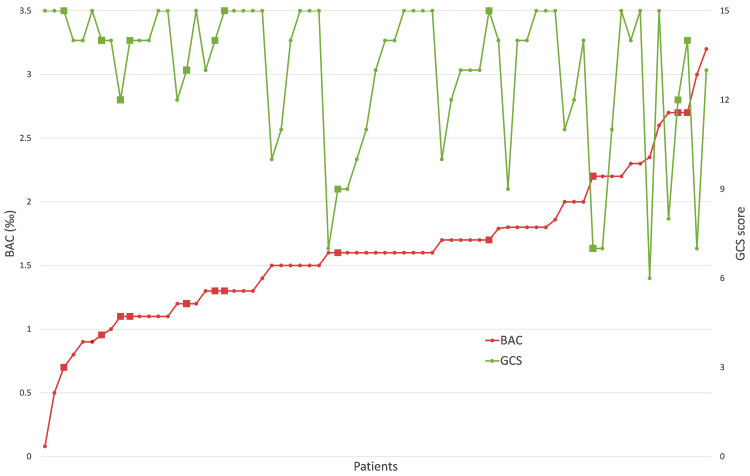
GCS and BAC levels of 71 patients in the study cohort. (BAC of 1 patient was missing). BAC levels are depicted in red, GCS scores are shown in green. Squares indicate co-ingestion of drugs, as indicated by the patients or paramedics (12 patients)

BAC levels and GCS scores were obtained from the medical reports at the University Children’s Hospital and were between 0.08–3.20‰ (mean 1.63‰, median 1.60‰) and 6–15 (mean 13, median 14) as summarized for each individual patient in Table S1. In Fig. 1, individual GCS scores are shown with increasing BAC levels. While BAC and GCS levels show opposing trends, large inter-individual differences between the different patients were observed. However, the time relationships between alcohol consumption and blood sample collection were unknown, which could have affected the results.

**Fig. 2:**
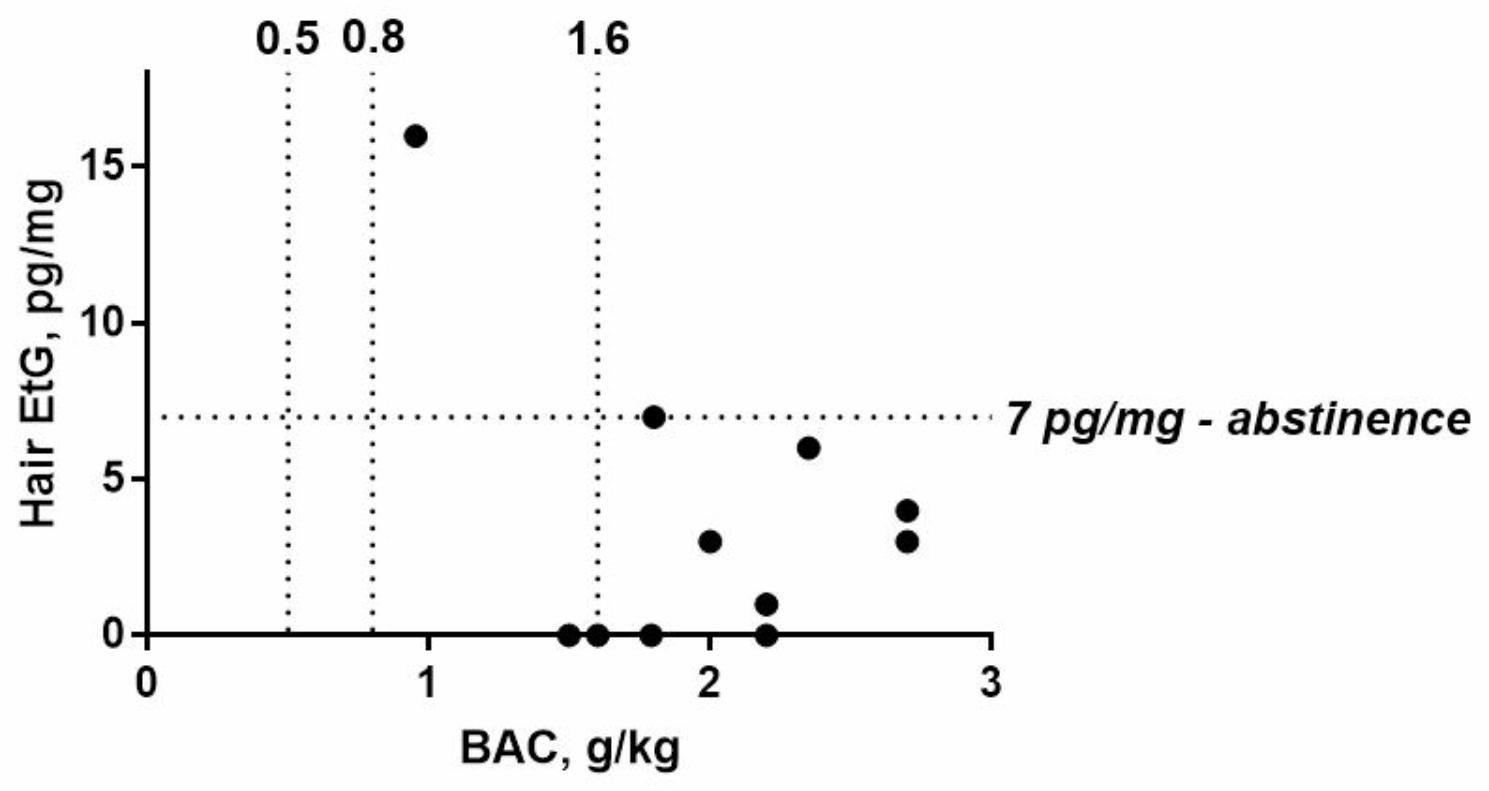
Relation between measured BAC (x-axis, g/kg) and EtG in hair (y-axis, pg/mg). The dotted line represents the commonly accepted cut-off value of 7 pg/mg for abstinence

Hair samples were available from 13 patients, and 11 samples were measured for EtG. In these 11 cases, BAC levels ranged from 0.954–2.7‰ (mean 1.98‰, median 2.00‰), while EtG concentrations ranged from undetectable to 16 pg/mg (mean 3.64 pg/mg, median 3.00 pg/mg) as shown in Table S1. In general, a moderate concordance between BAC levels and the degree of impairment was found, especially when alcohol was consumed regularly [39]. The actual measured BAC is always dependent on the timeframe of the last alcohol intake, which often remains unknown. Also, a comparison of the analytical BAC levels compared to the self-declared amount of drinks cannot be reliably calculated, as the patient’s drinking information remains vague (Table S1). Specific information on the kind, amount and time of drinking would have been necessary for a reliable, theoretical blood alcohol calculation. In our study cohort, nine out of the 11 patients with available blood and hair samples had BAC levels above 1.6‰. In their recent consensus on alcohol markers in hair [40], the Society of Hair Testing provided three criteria for classifying EtG concentrations in hair: (1) a concentration of 5 pg/mg or less EtG in the proximal head hair segment with a length of 3–6 cm does not contradict self-reported abstinence. (2) a concentration greater than 5 pg/mg EtG in the proximal head hair segment with a length of 3–6 cm strongly suggests repeated alcohol consumption. (3) a concentration greater than or equal to 30 pg/mg EtG in the proximal head hair segment with a length of 3–6 cm strongly suggests chronic excessive alcohol consumption. Based on this classification, eight cases fell into the first category, and three cases fell into the second category, two of which just exceed the recently proposed cut-off of 5 pg/mg (the former cut-off was 7 pg/m). None of the measured cases provided EtG hair results, indicating chronic excessive alcohol consumption. Figure 2 graphically illustrates the relation between BAC and EtG in this small sub-cohort. It is apparent that the majority of cases exhibited high BAC but low EtG concentrations, which is consistent with a binge drinking effect, despite the small dataset. The only outlier is represented by case 10, whose EtG concentration in hair (16 pg/mg) indicated repeated alcohol drinking, but no chronic excessive alcohol consumption. According to patient information, alcohol is regularly consumed in form of three to four drinks once a month.

### Results from drug screening analysis in blood and hair samples

Blood samples are considered the matrix of choice for classifying drug concentrations as therapeutic (expected/desired drug action), sub-therapeutic (no drug action), toxic (above intended drug doses, most likely associated with (harmful) side effects), or even lethal. This allows for the interpretation of impairments at certain time points [41]. The analysis of keratinized matrices such as hair has received considerable attention in clinical and forensic toxicology as they are known to accumulate various substances over time (months to years) [42, 43]. Keratinized matrices provide long-term information regarding the consumption of substances (drugs, illicit drugs, or pharmaceuticals) or exposure to substances. The combined analysis of classical matrices (typically blood or urine) and keratinized matrices provides beneficial information on short-term (e.g. acute) and long-term (chronic) substance use.

**Fig. 3:**
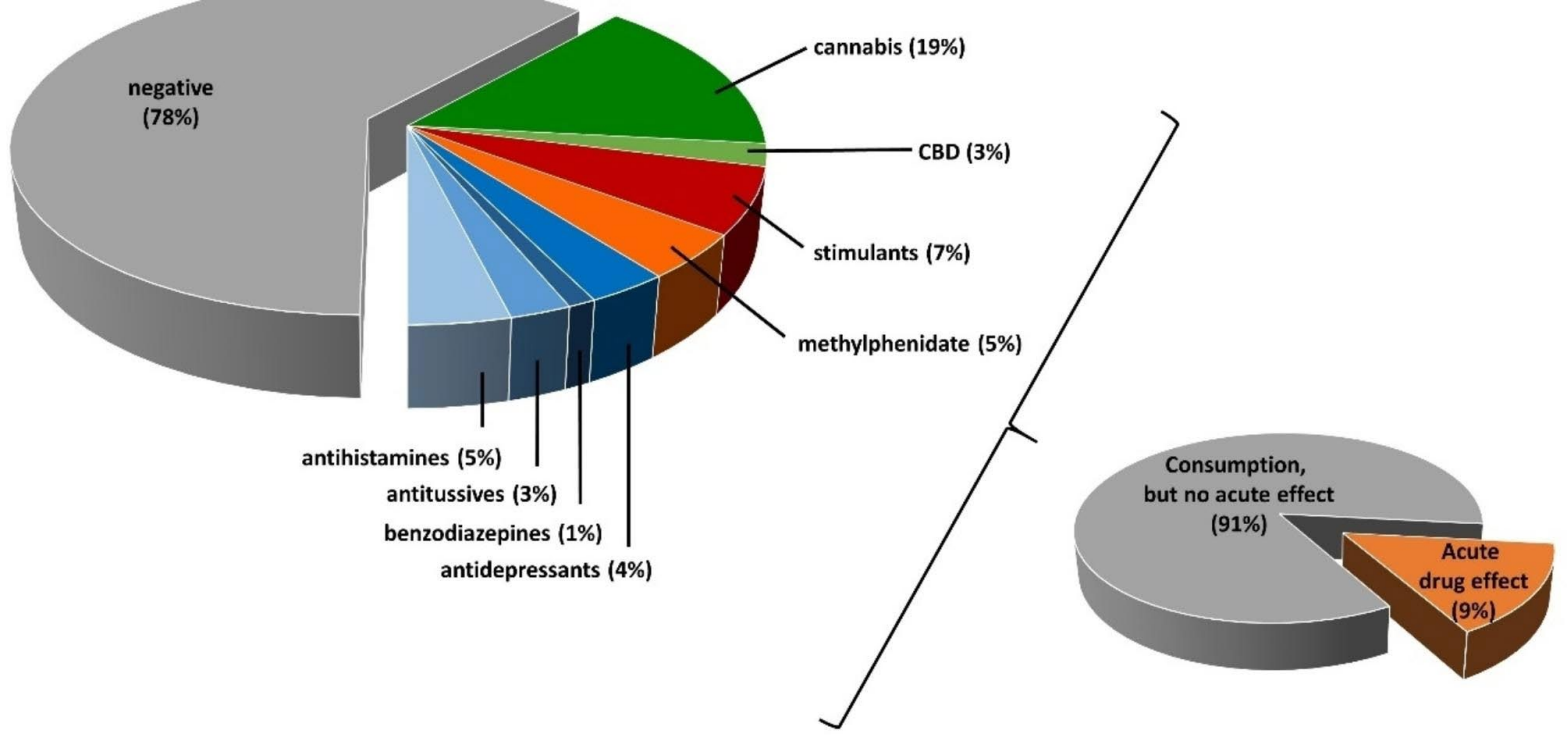
Drug consumption – results from blood and hair analysis. “negative” includes negative results (except for ethanol) and typical emergency drugs or analgesics. One case can be positive for several substances, therefore all data together do not add up to 100%

Blood and hair samples from 72 and 13 patients, respectively, were screened for drugs. Cases were categorized as negative if no drug was detected in either the blood or hair samples. Cases where drugs were detected in either sample were categorized as positive. Multiple drug detection was possible in some cases. Table 1; Fig. 3 provide an overview of the results.

**Table 1:**
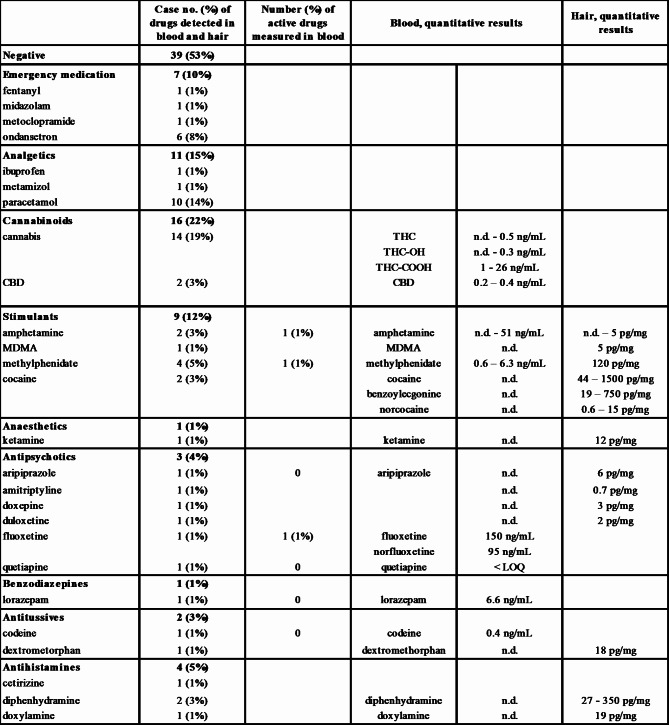
Summary of detected drug intake in blood and hair samples

In total, 39 cases (53%) showed negative results (except for ethanol), while 18 cases (25%) had only typical emergency drugs (midazolam, ondansetron, metoclopramide) or analgesics (ibuprofen, paracetamol, metamizol) detected, which were deemed irrelevant from a toxicological point of view. Cannabis was the most commonly detected drug of abuse, with THC-rich cannabis found in 14 cases, and CBD-cannabis in three cases, respectively. In Switzerland, cannabis products with THC levels below 1%, the so-called CBD-cannabis have been legal since 2017. Stimulants, including the prescription drug (dex)methylphenidate, constituted the second most commonly detected drug class, with a total of nine positive cases. Of these, four cases tested positive for methylphenidate, two for amphetamine, two for cocaine, and one for MDMA. Methylphenidate was detected in the blood at sub therapeutic to therapeutic levels and in one available hair specimen. The concentration in hair fell in the medium range compared to our reference values, indicating repeated or regular use of methylphenidate, consistent with the blood results and self-reported ADHD diagnosis and Concerta® prescription. Effective concentrations of amphetamine were detected in the blood of case 81, but no hair sample was available. The chosen analytical methodology could not differentiate between (illegal) street amphetamine intake and the prescription drug for ADHS, lisdexamphetamine, metabolized to amphetamine in the body. However, according to the patient information, none of the patients received lisdexamphetamine. In three other cases, the intake of amphetamine, cocaine, and MDMA was only identified in hair samples, indicating a long time ago intake. Overall, the hair concentration of MDMA, amphetamine, and cocaine in two of these cases were very low (below the proposed SoHT cut-off), indicating a single or sporadic intake over the investigated time period (3–5 months before hair sampling). Cocaine and metabolite concentrations only in case 32 were in a medium range compared with our in-house reference values, indicating repeated cocaine use over the investigated time period. Other detected drugs included antihistamines (n = 4), antidepressants and antipsychotics (n = 3), antitussives (n = 2), and benzodiazepines (n = 1). Patient 36 had therapeutic fluoxetine and sub-therapeutic quetiapine blood concentrations, and patient 37 had sub-therapeutic to weak therapeutic lorazepam blood concentrations. Four different antipsychotics were found in the 13 analyzed hair samples from different cases, namely aripiprazole, amitriptyline, duloxetine, and doxepine, with concentrations falling within a very low range, indicating a very weak or single intake, or even past intake (grow-out effect). Diphenhydramine concentrations in case 32 were in the high range compared to our reference values, indicating repeated intake, but the simultaneous negative results in the blood excluded an acute effect. Overall, out of the 37 cases positive for any drug, only three cases had an acute drug influence at the time of blood sampling.

The overall statements of the patients on co-ingestion of drugs matched the analytical findings well. Nine patients indicated cannabis consumption, while 14 tested positive for cannabis, with no or low THC detection as the psychoactive drug. Three patients reported a prescription use of methylphenidate, that was proven in these three and one additional hair sample. Given the long detection windows for drugs in hair, the detection of methylphenidate does not necessarily indicate its use at the time of hospitalization. One other patient reported a therapy with an antidepressant and a sedative, with toxicological analysis revealing active concentrations of fluoxetine and previous use of quetiapine. One participant admitted to using liquid ecstasy (gamma-hydroxybutyric acid, GHB), however, GHB was not covered by the applied methods. In one case, paramedics reported dormicum (midazolam) application during treatment. None of the other patients admitted to using illegal drugs, such as amphetamine or cocaine, other than cannabis. However, previous consumption of cocaine, MDMA, and amphetamine has been proven. Binge drinking is associated with drug use, mainly stimulants and cannabis [44]. An analysis of 170’000 US youth and adults found that binge drinkers were twice as likely to consume drugs compared to non-binge drinkers and four times as likely to consume drugs compared to non-drinkers.

Overall, the lack of a matching control cohort, the small sample size and patient acceptance problems for hair collection made it difficult to conduct a comprehensive evaluation of substance (mis)use.

### Results from VNTR analysis

The initial idea was to investigate the correlation between the long and short versions of 5-HTTLPR and the level of tolerance. From the initial 72 samples 26 had to be excluded due to drug co-ingestion (based on self-reporting and blood concentrations) or non-European ethnic background, leaving 46 samples for further analysis. Among these were 9 individuals with high BAC (≥ 2‰) and 9 individuals with low GCS (score ≤ 10), but there were not enough individuals to build meaningful low LR (high BAC / high GCS) and high LR groups (low BAC / low GCS), with 6 patients each. The results of HTTLPR genotyping are provided in Table S1. However, owing to the small sample size and diverse genetic background, statistical analysis was not possible, and no conclusions could be drawn. Nevertheless, we believe that it is relevant to report the genotypes obtained for each patient (Table S1). Notably, we identified the XL allele in a patient with an African background. The XL variant is most frequently found in individuals of Asian and African origin [21].

### Limitations of the study

Our study has some limitations. The patients self-reported alcohol consumption was sometimes vague and we have refrained from converting this into number of standard drinks, as stated in the definition of binge drinking. However, all patients had symptoms of an alcohol intoxication and could be considered as binge drinkers. Only 72 patients could be included from which only 13 agreed to provide a hair sample in addition to blood. The study missed a matching control group of none-suspected binge drinkers and only investigated patients once, after admission to the emergency department, rather than continuously over longer time periods. One aim of the study was to evaluate hair analysis to assess binge drinking. As only 13 hair samples could be sampled and only 11 could be used for EtG analysis the outcome of the results is not significant. Nevertheless, as expected EtG levels were low in binge drinkers which confirms that EtG in hair is more suitable to access chronic excessive drinking over a longer time period rather than to assess occasional binge drinking. Other alcohol markers like phosphatidylethanol (PEth) might be more suitable to assess binge drinking effects with blood analysis. EtG and PEth testing are nowadays considered as complementary tools in ethanol (abstinence) control testing, covering different time windows of ethanol consumption and provide different levels of sensitivity. At the time of patient enrollments (2014–2019), PEth was not yet a routinely used method in general and not implemented in our laboratory. At that time, EtG – in our opinion – could be considered as the best marker for ethanol consumption behavior, although it is well known, that binge drinking might not be properly reflected in hair EtG. This was once again confirmed in our sample cohort (Fig. 2). However, unfortunately, no dried blood spots were initially collected, and we would not consider the analysis of PEth in whole blood stored at -20 ° C for more than one year to be reliable, giving its stability issues.

## Conclusions

We comprehensively analyzed the drinking behavior of adolescents who engage in binge drinking, on the basis of a particular drinking event, as well as their underlying medical conditions and (prescribed) drug consumption. In contrast to other studies, we provide analytical evidence on alcohol and drug consumption of young people and thereby complement epidemiologic evaluations and surveys. In our cohort of 72 binge drinking patients, blood alcohol concentrations ranged from 0.08–3.20‰. In almost half of the cases other drugs were consumed in addition, mostly cannabis and stimulants. Compliance between patients’ statements and measured substances matched well. However, we could not investigate the potential role of the 5-HTTLPR polymorphism as a marker for innate alcohol tolerance, due to the diverse genetic background of the cohort and the small sample size. We anticipate that our study will enhance understanding of youth consumption behavior and to support ongoing efforts to address the harmful effects of binge drinking and substance use.

### Electronic supplementary material

Below is the link to the electronic supplementary material.

Supplementary Material 1

Supplementary Material 2

## Data Availability

All data generated or analyzed during this study are included in this published article and its supplementary information files.

## Abbreviations

AUD: Alcohol Use Disorder
BAC: Blood alcohol concentrations
EtG: Ethyl glucuronide
GCS: Glasgow Coma Scale
VNTR: Variable number of tandem repeats
