# Supplementary material for: Analytical description of adolescent binge drinking patients
Source: BMC Pediatr. 2023 Oct 16;23:512. doi: 10.1186/s12887-023-04325-2 (PMC10577939; doi:10.1186/s12887-023-04325-2)
Supplement: Supplementary file 2 — Supplementary Material 2 [file 12887_2023_4325_MOESM2_ESM.pdf]

Supplementary Material: Details on the analytical methods

Sample preparation of whole blood samples for screening and quantitative analysis

KF-stabilized whole blood samples were extracted using protein precipitation (PP) with slight modifications. Briefly, to 200 µL whole blood, a heavy-labelled internal standard (IS) or an IS-mixture was added, and PP was performed by slowly adding acetonitrile (400 – 600 µL depending on the method) during vortexing. The mixture was vortexed, shaken for 10 min, and centrifuged (5 minat 10,000 rpm). Different volumes of the supernatant (250 – 550 µL) were transferred into an auto sampler vial. After the addition of formic acid (20%, v/v; 20 – 50 µL), the supernatant was evaporated to dryness under a gentle stream of nitrogen at ambient temperature and reconstituted in varying amounts (50 - 300 µL) of a mixture of mobile phases A and B. Subsequently, three separate analyses were conducted: untargeted screening analysis, confirmative, quantitative analysis of a multitude of drugs, and identification and quantification of cannabis, similar to a previous study [1].

Whole Blood (qualitative) screening by LC-MS/MS

Extracted blood samples were analyzed using an untargeted data acquisition approach with a Toxtyper® LC-MS/MS system. The system was composed of an UltiMate 3000 LC system controlled by Chromeleon 6.80 software (Thermo Fischer Scientific, Reinach, Switzerland) and an Amazon Speed^{TM} ion trap mass spectrometer (Bruker Daltonik GmbH, Bremen, Germany) equipped with a standard electrospray ionization (ESI)-source. Separation was achieved using an Acclaim^{TM} RSLC 120 C18 column (2.1 x 100 mm, 2.2 µm, Thermo Fisher Scientific) at a flow rate of 0.5 mL/min and an injection volume of 5 µL. The gradient elution method was programmed as follows: 0–1 min 1% B; from 1–4 min to 21% B, from 4–8 min to 29% B, 8–13 min increase to 64% B and till 15 min to 99% B; hold for 2 min; decrease to starting conditions and re-equilibration for 2 min. The MS was operated in auto-MSn-mode (Ultrascan, m/z 70-800, 32500 m/z per second). A positive electrospray ionization mode with the following source parameters was applied, capillary voltage: 4500 V; end plate offset: 500 V; nebulizer gas: 7.3 psi; dry gas: 4.0 L/min; and dry temperature: 180°C. The trap ion charge control was set to 300000 and the maximal accumulation time was 100 ms. The collision-induced fragmentation (CID) threshold was 10000 units, the amplitude was set to 0.8 V. MS/MS spectra were triggered for the highest intensity ion, followed by exclusion after one occurrence. Data were automatically matched to the Maurer/Wissenbach/Weber database [2], and reports were

generated using Data Analysis 4.2 and Compass Open Access software (Bruker Daltonik GmbH).

Quantification of drugs in blood

Quantification of positive results from the blood screening analysis was performed by LC-MS/MS using a targeted in-house multianalyte method. The method covered 82 drugs or drug metabolites from various drug classes such as stimulants, opioids, benzodiazepines, antidepressants, antipsychotics, antitussives, and antihistamines. Limits of quantifications (LOQs) were as follows: 2.4 ng/mL, 6 ng/mL, 2.4 ng/mL, 2.4 ng/mL, 1.6 ng/mL, 8.0 ng/mL, 8.0 ng/mL, 20 ng/mL, 4.0 ng/mL, 2.0 ng/mL, and 16 ng/mL for amphetamine, benzoylecgonine, cocaine, dextromethorphan, diphenhydramine, doxylamine, ketamine, lorazepam, MDMA, methylphenidate, and quetiapine, respectively. Briefly, measurements were carried out on a Thermo Fischer Ultimate 3000 UHPLC system (Thermo Fischer Scientific) coupled to a Sciex 5500 QTrap linear ion trap quadrupole mass spectrometer (Sciex, Darmstadt, Germany). The LC settings were as follows: Phenomenex (Aschaffenburg, Germany) Kinetex PS C18 (2.6 µm, 100 A, 100x2.1 mm), gradient elution with 10 mM ammonium formate buffer in water containing 0.1 % (v/v) formic acid (pH 3.5, eluent A), and acetonitrile containing 0.1 % (v/v) formic acid (eluent B). Positive electrospray ionization (ESI) was used, and the MS was operated in scheduled multiple reaction monitoring (MRM) mode using two transitions for each analyte. MS was controlled by Analyst® 1.6.2 software (Sciex). Quantitation was performed using MultiQuant® 2.1.1 software (Sciex). The method was fully validated according to national and international guidelines [3-6]. Quantification was performed on the peak area ratios of drug to IS against an eight-point calibration covering an extended therapeutic range for prescription drugs and ranges typically observed in forensic toxicology for drugs of abuse. Each analysis batch was controlled by additional randomized measurements of three quality control samples.

Quantitative analysis for cannabinoids in blood

Given the undetectability of cannabinoids in the screening analysis, all blood samples were subjected to targeted cannabis analysis on tetrahydrocannabinol (THC), cannabidiol (CBD) and the THC metabolites hydroxy-THC (THC-OH) and THC carboxylic acid (THC-COOH). LOQs were as follows: 0.5 ng/mL, 0.2 ng/mL, 0.5 ng/mL, and 5 ng/mL for THC, CBD, THC-OH, and THC-CCOH, respectively. Sample extracts were analyzed on a Thermo Fischer Ultimate 3000 UHPLC system (Thermo Fischer Scientific) coupled to a Sciex 5500 QTrap linear ion trap

quadrupole mass spectrometer (Sciex). The LC settings were as follows: Phenomenex (Aschaffenburg, Germany) Kinetex C18 (2.6 µm, 100A, 50 x 2.1mm), gradient elution with 5 mM ammonium formate buffer in water containing 0.1 % (v/v) formic acid (eluent A) and methanol (eluent B); injection volume 10 µL. MS mode, QC, and data evaluation were performed as described above for the drug quantification.

Analysis of drugs, medication and ethylglucuronide in hair samples

Hair samples were first analyzed for drugs and medication to assess past drug exposure using a multianalyte approach by Scholz et al. [7]. Hair samples were washed with 15 mL water, 10 mL acetone, and 10 mL hexane. Each washing step was performed for 2 min, with slight shaking. The samples were dried and chopped into snippets using scissors. Approximately 20 mg of the snippets were exactly weighed into Eppendorf tubes and pulverized using a Retsch ball mill (Type MM 400, Retsch GmbH & Co. KG, Haan, Germany) by shaking in the presence of one tungsten carbide ball (Ø 7 mm, 3 g, Retsch) at 30 Hz for 10 min. After pulverization, 1.4 mL of methanol and 0.1 mL of an internal standard (IS) solution were added to each sample [7]. The Eppendorf tubes, together with the remaining tungsten carbide balls, were again placed in the ball mill and shaken at 10 Hz for 90 min. After centrifugation at 9000 rpm for 25 min, the supernatant was collected and dried at 35 °C under nitrogen. For the second micro-extraction step, 1 mL of 1-mM aqueous ammonium formate containing 0.1% formic acid/methanol (1:1, v/v) was added to the remaining hair powder, followed by shaking at 10 Hz for 90 min in a ball mill. The resulting supernatant was combined with the dried supernatant from the first step and dried at 35 °C under nitrogen. The dried residues were reconstituted in 150 µL methanol, vortexed, and 350 µL of a solution of 2-mM aqueous ammonium formate was added. The solutions were transferred to liquid chromatography (LC) vials. The LC–MS-MS system consisted of a Shimadzu Prominence high performance liquid chromatography system (Shimadzu, Duisburg, Germany) and a QTrap 5500 mass spectrometer (Sciex) using electrospray ionization (ESI) operating in positive mode. Separation was achieved using a Kinetex® F5 column (100 mm × 2.1 mm, 100 Å, 2.6 µm, Phenomenex) coupled with SecurityGuard™ ULTRA Cartridges ultra-high performance liquid chromatography (UHPLC) F5 (2.1 mm ID). Mobile phase A (water containing ammonium formate [1 mM] and formic acid [0.1%]) and mobile phase B (acetonitrile containing ammonium formate [1 mM] and formic acid [1 mM]) were used. A post-column spray of methanol was applied at a flow rate of 0.04 mL/min in order to support the ionization process, especially at the beginning of the gradient. The flow rate was set at 0.6 mL/min, the gradient was programmed as follows: 0.01

to 1.5 min, 3% eluent B; 1.5 to 9 min increasing to 60% eluent B; 9 to 10 min increasing to 95% eluent B; 10 to 11 min, 95% eluent B; 11 to 11.1 min decreasing to 3% eluent B, 11.1 to 12 min starting conditions (3% eluent B). The column oven temperature was set at 40°C. The dead time (t0) was about 0.3 min (0.19-mL void volume of the column).

In a second step hair samples that had enough material (> 5 mg) were analyzed for ethylglucuronide. EtG analysis (Limit of detection (LOD) 1 pg/mg and limit of quantification (LOQ) 2 pg/mg)) was performed following the protocol described by Binz et al. [8]. Briefly, the selected hair segment was washed twice with 15 ml of H_2O, followed by 15 ml acetone in a Sarstedt tube for 2 min. The hair samples were subsequently dried at room temperature and cut into small pieces of a few millimeters. Approximately 20 mg of hair were filled into an Eppendorf tube (2 ml) and pulverized with a single tungsten carbide ball in a ball mill. After pulverization, 1000 pg d5-EtG as internal standard and 1.5 ml of water were added, and samples were extracted twice for 15 min. Solid-phase extraction (SPE) was then performed using Waters Oasis1MAX cartridges. The cartridges were equilibrated using 2 ml of methanol and 2 ml of water. The supernatant from the samples was transferred to the cartridges, followed by 1 ml of water/ammonia (5%) solution and a washing step with 2 ml of methanol. The columns were then dried under vacuum for 5 min. Finally, the EtG was eluted with 2 ml of 2% formic acid in methanol. The eluate was evaporated under nitrogen at 35 °C using a metal heating block. The LC-MS system consisted of a Shimadzu Prominence XR high-pressure liquid-chromatography (HPLC) system coupled to a Sciex QTrap 5500 linear ion trap quadrupole mass spectrometer (Sciex). EtG was separated employing a Hypercarb TM PGC column (100 x 2.1 mm, 3 µm) with a guard column (10 x 2.1 mm, 3 µm) at 40 °C (Thermo Fisher Scientific). The mobile phase consisted of water with 1% ammonium (A) and acetonitrile (B), at a flow rate of 0.4 ml/min. The gradient was as follows: 0–5% B for 0–5 min, 5–90% B from 5 to 8 min, isocratic 90% B from 8 to 11 min, 90–0% B from 11t o 13 min, and an equilibration step of 1 min. The post column solvent addition of methanol was performed at 0.2 ml/min. The 5500 ion trap instrument was operated in negative ion mode with an ion-spray voltage of 4500 V, a source temperature of 600 °C, nitrogen at 20 psi as curtain gas, and nebulation and heating gases at 80 psi and 85 psi, respectively. The analytes were detected in multiple reaction monitoring mode (MRM), monitoring three transitions for EtG (221/75, 221/85 and 221/55) and two transitions for d5-EtG, (226/75 and 226/85). Analysis of the collected data was carried out using the Analyst software (version 1.6.2, Sciex). The collision-associated dissociation gas was set to ‘‘high’’. All source parameters were optimized under LC conditions and the electrical parameters were optimized by direct infusion.

References

1. Steuer AE, Eisenbeiss L, Kraemer T: Blood alcohol analysis alone versus comprehensive toxicological analysis - Systematic investigation of missed co-ingested other drugs in suspected alcohol-impaired drivers. *Forensic science international* 2016, 267:52-59.
2. Maurer HH, Wissenbach DK, Weber AA: Maurer / Wissenbach / Weber LC-MSn library of drugs, poisons, and their metabolites: Wiley-VCH; 2014.
3. Peters F, Hallbach J, Maurer H: Recommendations from the Society for Toxicological and Forensic Chemistry (GTFCh) concerning the validation of methods for toxicological analysis in the context of determining brain death. . *TIAFT Bulletin* 2005, 35(1):8-9.
4. Peters F, Drummer O, Musshoff F: Validation of new methods. *Forensic science international Genetics* 2007, 165(2-3):216-224.
5. Kruve A, Rebane R, Kipper K, Oldekop ML, Evard H, Herodes K, Ravio P, Leito I: Tutorial review on validation of liquid chromatography-mass spectrometry methods: part I. *Anal Chim Acta* 2015, 870:29-44.
6. Kruve A, Rebane R, Kipper K, Oldekop ML, Evard H, Herodes K, Ravio P, Leito I: Tutorial review on validation of liquid chromatography-mass spectrometry methods: part II. *Anal Chim Acta* 2015, 870:8-28.
7. Scholz C, Cabalzar J, Kraemer T, Baumgartner MR: A Comprehensive Multi-Analyte Method for Hair Analysis: Substance-Specific Quantification Ranges and Tool for Task-Oriented Data Evaluation. *J Anal Toxicol* 2021, 45(7):701-712.
8. Binz TM, Baumgartner MR, Kraemer T: The influence of cleansing shampoos on ethyl glucuronide concentration in hair analyzed with an optimized and validated LC-MS/MS method. *Forensic science international* 2014, 244:20-24.
